# Fixation of intracapsular fracture of the femoral neck using combined peripheral nerve blocks and transthoracic echocardiography in a patient with severe obstructive hypertrophic cardiomyopathy: a case report

**DOI:** 10.1186/s40981-019-0287-1

**Published:** 2019-10-22

**Authors:** Sho Yamazaki, Takeshi Omae, Keito Koh, Sonoko Sakuraba, Yosuke Katsuda, Masateru Kumemura

**Affiliations:** Department of Anesthesiology and Pain Clinic, Juntendo University Shizuoka Hospital, 1129 Nagaoka, Izunokuni, Shizuoka 410-2295 Japan

**Keywords:** Hypertrophic obstructive cardiomyopathy, Transthoracic echocardiography, Non-cardiac surgery, Regional anesthesia

## Abstract

**Background:**

Hypertrophic obstructive cardiomyopathy (HOCM) is a type of hypertrophic cardiomyopathy associated with left ventricular outflow tract stenosis. The increased pressure gradients across the left ventricular outflow tract in patients with HOCM could lead to circulatory collapse. We describe our experience with perioperative management under femoral nerve block (FNB), lateral femoral cutaneous nerve block (LFCNB), and transthoracic echocardiography (TTE) monitoring during open reduction and internal fixation of a femoral neck fracture in a patient with severe HOCM.

**Case presentation:**

A 72-year-old man, who was indicated to undergo open reduction and internal fixation of an intracapsular femoral neck fracture, had a history of treatment for hypertension and HOCM. He had heart failure for 4 years and was hospitalized several times. He was resuscitated after ventricular fibrillation and received an implantable cardioverter-defibrillator at that time. He also had severe physical limitations (New York Heart Association class III). We selected FNB and LFCNB as the methods for anesthesia and injected 0.25% levobupivacaine (20 mL) around the femoral nerve and 0.25% levobupivacaine (10 mL) into the lateral femoral nerve region. He underwent TTE during the perioperative period, which enabled us to perform hemodynamic and morphological evaluations of the heart. The intraoperative TTE findings remained stable from before the induction of anesthesia to the patient’s exit from the operating room. Postoperatively, his hemodynamic parameters continued to remain stable.

**Conclusions:**

In this case, FNB and LFCNB contributed to hemodynamic stability during non-cardiac surgery. Additionally, TTE was useful for the perioperative evaluation of cardiac hemodynamics and morphology in our patient with severe HOCM.

## Background

Hypertrophic cardiomyopathy (HCM) is defined as left or right ventricular hypertrophy without any apparent cause, such as hypertension or aortic stenosis [1, 2]. Hypertrophic obstructive cardiomyopathy (HOCM) is a type of HCM that involves left ventricular outflow tract obstruction (LVOTO) [1, 2]. The increased pressure gradient across the left ventricular outflow tract (LVOT) in the patients with HOCM could lead to circulatory collapse due to systolic anterior motion of the mitral valve (SAM) and mitral regurgitation (MR) [3]. Hemodynamic variables such as preload, afterload, and ventricular myocardial contractility are prone to fluctuations in the perioperative period. These circulatory changes would increase the pressure gradient across the LVOT [3–5]. Here, we report a case in which peripheral nerve block and monitoring with transthoracic echocardiography (TTE) was useful in the management of a patient with severe HOCM during osteosynthesis of a femoral neck fracture.

## Case presentation

A 72-year-old man (body weight, 50 kg; height, 158 cm) with a history of treatment for hypertension and HOCM visited our hospital with a chief complaint of pain in the buttock following a fall from his bed. Detailed examination revealed intracapsular fracture of the left femoral neck, for which open reduction and internal fixation (ORIF) was indicated. He was diagnosed with HOCM and LVOTO 6 years prior and was under treatment with carvedilol and cibenzoline. He had heart failure for 4 years and had been hospitalized several times because of exacerbation of LVOTO. Three years ago, he was resuscitated following ventricular fibrillation and received an implantable cardioverter-defibrillator (ICD) at that time. ICD activation was subsequently confirmed. Although the patient had no symptoms at rest before the injury, he had severe physical limitations [New York Heart Association (NYHA) Class III]. Cardiac auscultation revealed a Levine III/IV systolic murmur in the third intercostal space at the left sternal border.

Preoperatively, the patient’s brain natriuretic peptide level was elevated (548 pg/mL). Further, electrocardiography revealed atrial fibrillation, and TTE revealed asymmetric septal hypertrophy (ventricular septum [VS] thickness, 28 mm; left ventricular posterior wall [PW] thickness, 14 mm; VS/PW ratio, 2.0). His pressure gradient across the LVOT was 65 mmHg, and mild MR was observed. On the basis of these findings and test results, the patient was diagnosed as having severe HOCM.

We selected femoral nerve block (FNB) and lateral femoral cutaneous nerve block (LFCNB) for the administration of anesthesia and TTE for monitoring. Carvedilol and cibenzoline were administered through the day of surgery. The patient was monitored using a five-lead electrocardiogram and pulse oximeter after admission to the operating room. Continuous arterial pressure monitoring was performed using a catheter inserted in the left radial artery prior to regional anesthesia via FNB. TTE was performed using a sector array transducer (Philips S8-3; Philips, Amsterdam, The Netherlands) and Philips iE33. The left ventricular ejection fraction, left ventricular filling pressure (e/e′), and cardiac index of the aortic valve were measured (hemodynamic parameters) using continuous Doppler ultrasound. The pressure gradient across LVOT, left ventricular end-diastolic and end-systolic diameters, and effective regurgitant orifice area were also measured (morphological parameters).

A linear ultrasonic probe (HFL50 15–6 MHz; SonoSite Inc., Bothell, WA, USA) and an 18-gauge Contiplex Tuohy needle (B-Braun Ltd., Tochigi, Japan) were used for the left FNB: 0.25% levobupivacaine (20 mL) was injected around the left femoral nerve, and 0.25% levobupivacaine (10 mL) was injected into the left lateral femoral nerve region. Surgery was initiated after achieving anesthesia. No additional anesthesia was required intraoperatively, and the surgery was completed. The surgery duration was 45 min, and the anesthesia duration was 72 min. The total blood loss was 30 mL, and the fluid balance was + 190 mL. The intraoperative TTE findings remained stable from before the induction of anesthesia to the exit from the operating room (Tables 1 and 2).

**Table 1:** Hemodynamic variables after FNB and LFCNB

Variables	Baseline	Time after FNB (minutes)							
		0	10	20	30	40	50	60	End of surgery
MAP (mmHg)	73	72	75	77	74	78	74	73	76
HR (beats/min)	67	61	64	59	65	58	60	62	57

*FNB* femoral nerve block, *LFCNB* lateral femoral cutaneous nerve block, *MAP* mean arterial pressure, *HR* heart rate

**Table 2:** Changes in echocardiographic parameters after FNB and LFCNB

Variables	Baseline	Time after FNB (minutes)							
		0	10	20	30	40	50	60	End of surgery
LVPG (mmHg)	69	70	64	63	67	64	65	66	63
LVDd (mm)	34	33	34	33	34	34	34	33	34
LVDs (mm)	19	18	18	18	18	18	18	18	18
EROA (cm^2^)	0.18	0.18	0.16	0.16	0.17	0.16	0.16	0.16	0.16
EF (%)	66	68	63	63	66	63	64	64	63
e/e′	17	18	17	17	17	16	17	17	16
CI (L/min/m^2^)	2.4	2.2	2.4	2.4	2.3	2.4	2.4	2.3	2.4

*FNB* femoral nerve block, *LFCNB* lateral femoral cutaneous nerve block, *LVPG* left ventricular pressure gradient, *LVDd* left ventricular dimension at diastole, *LVDs* left ventricular dimension at systole, *EROA* effective regurgitant orifice area, *EF* ejection fraction ratio (%), determined using the modified Simpson method, *e/e′* color tissue Doppler imaging loops were obtained in the apical four-chamber view, and peak early diastolic velocity (e′) was measured at the base of the septum, *CI* cardiac index, determined using transthoracic echocardiography with continuous Doppler measurements in the aortic valve

Postoperatively, the patient was transferred to the intensive care unit (ICU) and the hemodynamic parameters remained stable. Acetaminophen 1000 mg was administered intravenously 10 h after admission to the ICU for postoperative pain, after which there was no complaint of pain. His hemodynamic parameters continued to remain stable, and he was discharged from the ICU on the following day.

## Discussion

In this report, we describe our experience with anesthesia management during ORIF of femoral neck fracture in a patient with severe HOCM. Intraoperatively, the patient was monitored using TTE.

Cases of HCM can be classified as hypertrophic nonobstructive cardiomyopathy, HOCM, apical HCM, mid-ventricular HOCM, and dilated-phase HCM [4, 6]. Additionally, patients who satisfy any of the following criteria are diagnosed as having a severe disease: moderate-to-severe restriction of physical activity (NYHA Class III–IV), a history of sustained ventricular tachycardia or ventricular fibrillation, and a history of hospitalization for heart failure or arrhythmia treatment. The patient in this case had been diagnosed as having HOCM previously on the basis of LVOT stenosis, MR, and SAM. Additionally, he had developed ventricular fibrillation, for which an ICD was subsequently implanted, and his physical activity was moderately or severely restricted. Therefore, he was diagnosed as having severe HOCM [4].

Although general or spinal anesthesia is usually selected for ORIF of hip fracture, both methods can cause hypotension in approximately one-third of patients [7]. By contrast, FNB, which has less of an effect on the sympathetic nerves, is expected to impact blood pressure to a lesser extent than spinal anesthesia [8–10]. Notably, decreased systemic vascular resistance due to anesthesia is associated with a high risk of an increased pressure gradient across the LVOT, which may create a stronger Venturi effect [4, 5, 11]. This could cause aspiration of the anterior mitral leaflet into the LVOT, thus worsening MR [4, 5, 11]. Our patient had HOCM with a pressure gradient > 50 mmHg across the LVOT. Therefore, we selected FNB and LFCNB for regional anesthesia to reduce the effect on systemic vascular resistance. However, insufficient anesthesia may enhance sympathetic nervous system activity, which would elevate the risk of increased cardiac contractility. This, in turn, could increase the pressure gradient across the LVOT and cause hemodynamic decompression [4, 5, 11]. To account for this risk, we did not initiate surgery in our patient until we had confirmed the achievement of sufficient anesthesia from the FNB and LFCNB. Hemodynamics were stable during the surgery (Tables 1 and 2).

To our knowledge, this is the first report of peripheral nerve block under monitoring with TTE in a patient with HOCM. We subjected our patient to continuous arterial pressure measurement and TTE in addition to the monitoring procedures described in the guidelines of the Japanese Society of Anesthesiology. Although the pulmonary catheter may be useful for circulatory management [12], recent studies point out the invasiveness of this device and the associated risk factors that may worsen patient outcome [13–15]. Therefore, we selected TTE for circulatory management in our case. The echocardiographic cardiac output measurements have been reported to be as accurate as thermal dilution according to assessment using a pulmonary arterial catheter, which is the current gold standard for cardiac output measurement [16, 17]. As TTE could enable evaluation of cardiac hemodynamics and morphology, we could diagnose and treat the condition in a timely manner despite the increase in the pressure gradient at the LVOT site or worsening of MR. Although we had evaluated the systolic and diastolic functions, pressure gradient at the LVOT, and degree of MR, these parameters remained unchanged during the surgery (Table 2).

Our patient had previously undergone ICD implantation. The ICD was disabled to avoid malfunction. An external defibrillator was available for use in the event of ventricular tachycardia or ventricular fibrillation [18, 19].

Postoperative pain could increase sympathetic nervous system activity, which could increase the pressure gradient across the LVOT. The combined peripheral nerve blocks contributed to postoperative cardiovascular stability and pain relief. In the early postoperative period, pain relief can be achieved with the peripheral nerve blocks supplemented by non-opioid drugs.

## Conclusions

We selected a combination of FNB and LFCNB to maintain the intraoperative preload, afterload, and ventricular myocardial contractility and TTE to evaluate the hemodynamic and morphological parameters of non-cardiac surgery in a patient with severe HOCM. The patient’s hemodynamic parameters were stable, and there were no complications in the perioperative period. Our findings suggest that FNB could reduce hemodynamic changes during non-cardiac surgery and TTE could contribute to the perioperative evaluation of cardiac hemodynamics and morphology in a patient with severe HOCM.

## Data Availability

The data in this case report are available from the corresponding author on reasonable request.

## Abbreviations

FNB: Femoral nerve block
HCM: Hypertrophic cardiomyopathy
HOCM: Hypertrophic obstructive cardiomyopathy
ICD: Implantable cardioverter-defibrillator
ICU: Intensive care unit
LFCNB: Lateral femoral cutaneous nerve block
LVOT: Left ventricular outflow tract
LVOTO: Left ventricular outflow tract obstruction
MR: Mitral regurgitation
NYHA: New York Heart Association
ORIF: Open reduction and internal fixation
PW: Posterior wall
SAM: Systolic anterior motion of the mitral valve
TTE: Transthoracic echocardiography
VS: Ventricular septum
